# Rice Bran Supplementation Ameliorates Gut Dysbiosis and Muscle Atrophy in Ovariectomized Mice Fed with a High-Fat Diet

**DOI:** 10.3390/nu15163514

**Published:** 2023-08-09

**Authors:** Pei-Xin Huang, Chiu-Li Yeh, Suh-Ching Yang, Hitoshi Shirakawa, Chao-Lin Chang, Li-Hsin Chen, Yen-Shuo Chiu, Wan-Chun Chiu

**Affiliations:** 1 School of Nutrition and Health Sciences, College of Nutrition, Taipei Medical University, Taipei City 11031, Taiwan; zxc951s37@gmail.com (P.-X.H.); cly@tmu.edu.tw (C.-L.Y.); sokei@tmu.edu.tw (S.-C.Y.); lily85110011491@gmail.com (L.-H.C.); g556096005@tmu.edu.tw (Y.-S.C.); 2 Research Center of Geriatric Nutrition, College of Nutrition, Taipei Medical University, Taipei City 11031, Taiwan; 3 International Education and Research Center for Food Agricultural Immunology, Graduate School of Agricultural Science, Tohoku University, 468-1 Aramaki Aza Aoba, Aoba-ku, Sendai 980-8572, Japan; shirakah@tohoku.ac.jp; 4 Laboratory of Nutrition, Graduate School of Agricultural Science, Tohoku University, 468-1 Aramaki Aza Aoba, Aoba-ku, Sendai 980-8572, Japan; 5 Food Industry Research and Development Institute, Hsinchu 300193, Taiwan; chaolin4096@gmail.com; 6 Department of Orthopedics, Shuang Ho Hospital, Taipei Medical University, New Taipei City 23561, Taiwan; 7 Department of Nutrition, Wan Fang Hospital, Taipei Medical University, Taipei City 11696, Taiwan

**Keywords:** rice bran, menopause, high-fat diet, muscle atrophy, microbiota

## Abstract

Rice bran, a byproduct of rice milling, is rich in fiber and phytochemicals and confers several health benefits. However, its effects on gut microbiota and obesity-related muscle atrophy in postmenopausal status remain unclear. In this study, we investigated the effects of rice bran on gut microbiota, muscle synthesis, and breakdown pathways in estrogen-deficient ovariectomized (OVX) mice receiving a high-fat diet (HFD). ICR female mice were divided into five groups: sham, OVX mice receiving control diet (OC); OVX mice receiving HFD (OH); OVX mice receiving control diet and rice bran (OR); and OVX mice receiving HFD and rice bran (OHR). After twelve weeks, relative muscle mass and grip strength were high in rice bran diet groups. IL-6, TNF-α, MuRf-1, and atrogin-1 expression levels were lower, and Myog and GLUT4 were higher in the OHR group. Rice bran upregulated the expression of occludin and ZO-1 (gut tight junction proteins). The abundance of *Akkermansiaceae* in the cecum was relatively high in the OHR group. Our finding revealed that rice bran supplementation ameliorated gut barrier dysfunction and gut dysbiosis and also maintained muscle mass by downregulating the expression of MuRf-1 and atrogin-1 (muscle atrophy-related factors) in HFD-fed OVX mice.

## 1. Introduction

Postmenopausal women are at high risk of obesity [1], abdominal obesity, and metabolic syndrome because of hormonal changes, which rapidly increase fat mass and redistribute and accumulate fat in the abdomen [2,3,4]. These phenomena may lead to postmenopausal gut dysbiosis and increased muscle atrophy. Obesity increases muscle loss and dysfunction, termed as sarcopenic obesity. It increases the inflammatory response that causes muscle atrophy and insulin resistance. These problems can reduce the life quality of the elderly [5].

Myog is a specific transcription factor of the myogenic regulatory factors family. This transcription factor can upregulate the expression insulin-like growth factor (IGF)-1, which is secreted by the liver, and leads to the activation of the phosphoinositide 3-kinase/protein kinase B/mammalian target of rapamycin pathway to promote muscle synthesis [6]. Myostatin is a negative regulatory factor of skeletal muscle mass, it inhibits the activation of the aforementioned pathway and induces the expression of muscular atrophy-related biomarkers, such as MuRF-1 and Atrogin-1, thus causing muscle atrophy [7]. Interleukin (IL)-6 and tumor necrosis factor (TNF)-α are the proinflammatory cytokines that can upregulate muscle atrophy-related gene expression [8,9]. These metabolic imbalances may induce muscle atrophy in postmenopausal women with obesity.

Scholars have proposed the concept of the gut–muscle axis, which suggests that gut microbiota affect the strength and function of skeletal muscle [10,11,12]. Aging and the intake of a high-fat diet may disrupt the gut cell tight junctions (TJs); the downregulating of certain TJ proteins, such as occludin, and zonnula occludens (ZO)-1, may increase leaky gut and inflammatory cytokine secretion [13], resulting in obesity and impaired insulin resistance.

Short-chain fatty acids (SCFAs, e.g., acetate, propionate, and butyrate), are the primary metabolites produced by intestinal bacteria through the reaction of anaerobic fermentation from dietary fiber. SCFAs play key roles in gut microbiota–muscle crosstalk. For example, butyric acid helps prevent muscle mass loss and anti-inflammatory effects [14], increasing ATP production and inhibiting muscle breakdown and apoptosis [15].

Rice is an important food crop; it serves as an essential food for approximately 50% of the world’s people. The outer layer of rice is rice bran, a byproduct produced by the milling process. Rice bran is rich in macronutrients (carbohydrates, proteins, and lipids) and several micronutrients (P, Ca, Mg, K, and Mn). It also contains vitamins, dietary fiber, and several bioactive components such as γ-oryzanol and phenolic acid (the most abundant of these is ferulic acid) [16]. The dietary fiber and γ-oryzanol in rice bran are widely used to treat diabetes, obesity, and cancer [17,18,19]. The beneficial characteristics of rice bran are partially redound to its capability to transform the gut microbiome and help the host to restore and maintain the balance of microbiota [18]. However, to date, no study has focused on the effect of HFD and rice bran intake on gut microbiota and muscle atrophy under menopausal conditions. Therefore, we established an HFD-fed menopause mouse model to simulate obesity and metabolic disorders in menopausal women. We investigated whether rice bran supplementation reduces muscle loss and ameliorates gut dysbiosis in HFD-fed ovariectomized (OVX) mice and investigated the underlying mechanisms.

## 2. Materials and Methods

### 2.1. Animals and Experiment Design

Female ICR mice, aged 24–28 weeks, were purchased from BioLASCO (Yilan, Taiwan) and maintained in an animal room with a temperature of 24 ± 2 °C, humidity at 60% ± 5%, and a 12 h light/dark cycle. All mice had ad libitum access to a maintenance diet (AIN-93M) and distilled water during one week of acclimation. The animal procedures of this study were ethically conducted and agreed upon by the Institutional Animal Care and Use Committee of Taipei Medical University (protocol approval number: LAC-2019-0175).

After acclimation, all mice were arranged to receive an ovariectomy or sham surgery. Before surgery, the mice were subjected to anesthetization through intraperitoneal injection of 40 mg/kg Zoletil 50 (Virbac, Carros, France) plus 20 mg/kg Rompun (Bayer, Germany). All mice were assigned to five groups: sham; OVX mice receiving a control diet (OC); OVX mice receiving HFD (OH); OVX mice receiving control diet and rice bran (OR); and OVX mice receiving HFD and rice bran (OHR) groups The experimental diets were modified from the AIN93M diet (Table S1). The lipid content in the diet of the sham and OC groups was 9.5%, the proportions of protein, lipid, and carbohydrate in the diet of HFD were 12%, 40%, and 48%, respectively. For rice bran groups, 10% (by weight) semi-defatted rice bran (mixed from Koshihikari rice and Hitomebore rice, Sendi, Japan) was added to the diet formula. The proportion of carbohydrates, protein, fat, and dietary fiber was 22%, 18.5%, 9.7%, and 34.6%, respectively. For bioactive compounds analysis, free ferulic acid in the rice bran was extracted with methanol and analyzed using a Shimadzu UFLC Ultra-Fast Liquid Chromatography (Shimadzu Corporation, Kyoto, Japan). Operating conditions were as follows: column, COSMOSIL 5C18-AR-II Packed Column (4.6 mm I.D. × 250 mm, 5 μm, Nacalai Tesque, San Diego, CA, USA); the condition of mobile phase A was 1% (*v*/*v*) acetic acid/deionized water; mobile phase B was using 1% (*v*/*v*) acetic acid prepared using deionized water; the flow rate was set at 1 mL/min; the temperature of column was controlled at 37 °C. The lipid extract from rice bran was performed using a method reported by Suzuki et al. [20]. Then, the oryzanol content in rice bran oil was analyzed using a method modified by Xu et al. [21]. The concentrations of total polyphenol, ferulic acid, and γ-oryzanol were 5.5 mg/g, 115 μg/g, and 119.1 μg/g, respectively. The mice were fed with an experimental diet for twelve weeks. In the eleventh week, mice feces were collected continuously for seven days. After twelve weeks of experimental diet intervention, all mice were euthanized. Blood, liver, fat, muscle, colon, and cecum specimens were collected and stored at −80 °C in a freezer until further analysis.

### 2.2. Grip Strength Measurement

The grip strength of the forelimbs and all four limbs of the mice was determined using a grip strength meter (BIOSEB’s Grip Strength, Pinellas Park, FL, USA) before the intervention and in the sixth and twelfth weeks. The data were performed by means of the grip strength meter.

### 2.3. Intraperitoneal Glucose Tolerance Test

The intraperitoneal glucose tolerance (IPGTT) was measured 3 days before euthanasia after the mice were starved for 12 h. The mice were intraperitoneally administered with 2 g/kg glucose. Blood glucose was collected by needle at 0, 30, 60, 90, and 120 min. The measurement of glucose level at each time point was using a glucometer. (catalog number: TD-4207, EasiCheck, New Taipei City, Taiwan).

### 2.4. Blood Biochemical Analysis

Serum estradiol level was evaluated using the Mouse/Rat Estradiol ELISA Kit (catalog number: ES180S, Calbiotech, El Cajon, CA, USA). The levels of blood glucose, total cholesterol (TC), triglyceride (TG), alanine aminotransferase (ALT), aspartate aminotransferase (AST), blood urea nitrogen (BUN) were performed using an automatic blood analyzer (LEZEN Reference Lab, Taipei Taiwan). The level of plasma insulin was determined using the Mouse Insulin commercial ELISA kit (catalog number: 10-1249-01, Mercodia, Uppsala, Sweden). To estimate insulin resistance, the homeostasis model assessment-estimated insulin resistance (HOMA-IR) value was calculated as follows: fasting blood glucose level (mg/dL) × fasting insulin level (mIU/L)/405.

### 2.5. Cecum Microbiota Analysis

The EasyPrep Stool Genomic DNA Kit (catalog number: DPT-BC28, TOOLS, Taipei, Taiwan) was used to extract DNA fragments from the cecum contents (0.2 g) of the mice. The samples were sequenced with on Illumina MiSeq Plateform (San Diego, CA, USA). Bioinformatics analyses of the 16S rRNA sequence data were performed to identify the bacterial species in the samples (the data were performed by Tools Microbiology Research Center, Taipei, Taiwan).

### 2.6. Gastrocnemius Muscle and Colon Gene Expression

mRNA was extracted from 30 mg specimens of gastrocnemius muscle and colon by using the TRIzol reagent (catalog number: 15596018, Thermo Fisher Scientific, Waltham, MA, USA). The concentration and quality of RNA extraction were measured at 260/280 nm using a spectrophotometer (BioTek Gen5 Software (vsersion Gen 5) for Epoch). Subsequently, cDNA was synthesized from total RNA (2 μg) in the presence of reaction buffer, dNTPs, RNase inhibitor, and reverse transcriptase (catalog number: K1622, Thermo ScientificTM RevertAid First Strand cDNA Synthesis Kit, Waltham, MA, USA) final reaction volume: 20 μL. The polymerase chain reaction conditions were as follows: 42 °C for 60 min, followed by 70 °C for 5 min, and 4 °C for 5 min. The polymerase chain reaction was performed in duplicate by using the SYBR Green Master Mix (catalog number: K0221, Thermo Fisher Scientific Maxima SYBR Green/Rox qPCR Master [2X], Waltham, MA, USA) according to the manufacturer’s instructions. All primers (Table S2) were purchased from Mission Biotech (Taipei, Taiwan). The target genes’ expression was normalized against that of glyceraldehyde 3-phophate dehydrogenase. The relative expression levels of all target genes were performed using the comparative Ct method.

### 2.7. Fecal SCFA Concentrations

The fecal SCFAs extraction method was modified from a method described by Lottoli et al. [22]. Fresh feces samples were weighed and suspended in 1 mL of water containing 0.5% phosphoric acid per 0.1 g of sample; the suspension was collected and stored at −80 °C until further analysis. SCFAs were measured using gas chromatographic–mass spectrometry (5977B and 7820A; Agilent Technologies, Santa Clara, CA, USA). A Nukol™ 30 m × 0.25 mm, df 0.25 μm capillary column (24107, Supelco, Bellefonte, PA, USA) was used for this. The sample (injection volume: 1 µL) was injected in the pulsed split mode and an injector was at 250 °C. The measurement performed under 90 °C at a rate of 15 °C/min increased the temperature to 150 °C, at a flow of rate 3 °C/min to 170 °C and at a rate of 50 °C/min until final temperature at 200 °C (the total time was 16.267 min); solvent delay was 3.2 min. SCFAs were identified on the basis of the retention times of standard compounds including acetate, propionate, and butyrate (CRM46975, Supelco, Bellefonte, PA, USA), and were quantified using MassHunter Quantitative Analysis software (Agilent Technologies, Santa Clara, CA, USA).

### 2.8. Statistical Analysis

Data are presented as mean ± standard error of the mean values (SEM). All statistical analyses were performed using GraphPad Prism 6. After normal distribution and homogeneity of the data were tested, Student’s *t*-test was performed to analyze the differences between sham and OC groups. The OVX groups were compared using one-way analysis of variance test, followed by Tukey’s post-hoc test. A *p*-value of less than 0.05 was considered statistically significant.

## 3. Results

### 3.1. Effect of Rice Bran on the General Characteristics of the Mice

#### 3.1.1. Body Weight

The final body weight of the mice and the level of weight gain were significantly higher in OC group than in the sham group. The OH group had the highest mean weight; however, body weight of the mice was significantly decreased in the OHR group. Moreover, weight gain was significantly lower in the OR and OHR groups than in the OC and OH groups, respectively (Table 1). The average weight gain percentage was 38.9% in the OC group and 59.5% in the OH group, respectively. Rice bran supplementation had a lower weight gain percentage in the OR group (19.3%) and the OHR group (30.6%). Therefore, rice bran supplementation was helpful in reducing weight gain in HFD-fed OVX mice.

#### 3.1.2. Food Intake and Energy Consumption

The average amount of food intake was significantly lower in the OC group than in the sham group. Furthermore, food intake was lower in OH and OHR groups than in the OC group, but no significant difference was observed between the OH and OHR groups. The average energy intake did not vary significantly between the OH, OR, and OHR groups (Table 1).

### 3.2. Relative Tissue Weight of Mice

Table 2 presents the results of the relative tissue weight of mice. The relative weight of the liver showed no significant difference between OC and sham groups but was was significantly lower in the OHR group than in the OH group. The relative weight of the perinephric adipose tissue was significantly higher in the OC group than in the sham group. The relative weight of uterus adipose tissue was significantly higher in the OH group than in the OC group. The HFD increased abdominal fat accumulation. The relative weight of the uterus was significantly higher and that of the muscle was significantly lower in the OC group than in the sham group. The relative muscle weight was significantly higher in the OR and OHR groups than in the OC and OH groups, respectively (Table 2). Therefore, rice bran supplementation helps retain muscle mass.

### 3.3. Blood Biochemical Profiles

#### 3.3.1. Plasma Biochemical Data

The plasma estradiol level was significantly lower in the OVX mice than the sham group. No significant difference was observed between the OC and sham groups in glucose, alanine aminotransferase, and aspartate aminotransferase levels. However, the plasma glucose level in the OR group was significantly lower than in the OC group, and compared to the OH group, the glucose of the OHR group was significantly decreased. Alanine aminotransferase activity was significantly lower in the OR group than in the OC group, and nonsignificantly lower in the OHR group. The TC and TG levels were significantly higher in the OC group than in the sham group and significantly lower in the OHR group than in the OH group. The levels of BUN were significantly lower in the OR and OHR than in the OC and OH group, respectively (Table 3).

#### 3.3.2. Results of IPGTT and Area under the Curve of Glucose

**Figure 1:**
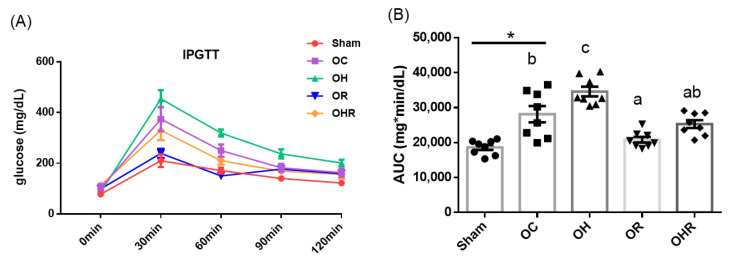
IPGTT results. (**A**) Variations in glucose levels during the IPGTT. (**B**) The AUC corresponds to glucose levels. Data are presented as mean ± SEM (*n* = 8). * Significant differences between the OC and sham groups (*p* < 0.05). Different letters indicate significant differences between the OC, OH, OR, and OHR groups (*p* < 0.05).

The area under the curve (AUC) corresponding to glucose level in the OC group was significantly higher than the sham group, the OH group was significantly higher than the OC group, the OR group was significantly lower than the OC group, and the OHR group was significantly higher than the OH group. (Figure 1) These results implied that rice bran supplementation reduced the level of glucose resposne in HFD-fed OVX mice.

#### 3.3.3. Insulin Levels and HOMA-IR Values

**Figure 2:**
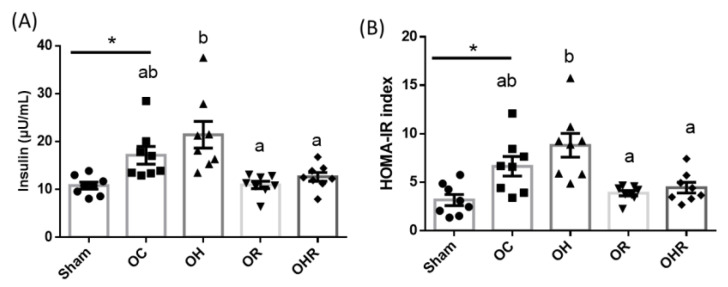
(**A**) Insulin (**B**) HOMA-IR values in the study groups. Data are presented as mean ± SEM (*n* = 8). * Significant differences between the OC and sham groups (*p* < 0.05). Different letters indicate significant differences between the OC, OH, OR, and OHR groups (*p* < 0.05).

The insulin levels and HOMA-IR values were significantly higher in the OC group than in the sham group and significantly lower in the OHR group than in the OH group (Figure 2). Therefore, rice bran supplementation reduced insulin levels and HOMA-IR values in the HFD-fed OVX mice.

### 3.4. The Grip Strength Test of Mice

**Figure 3:**
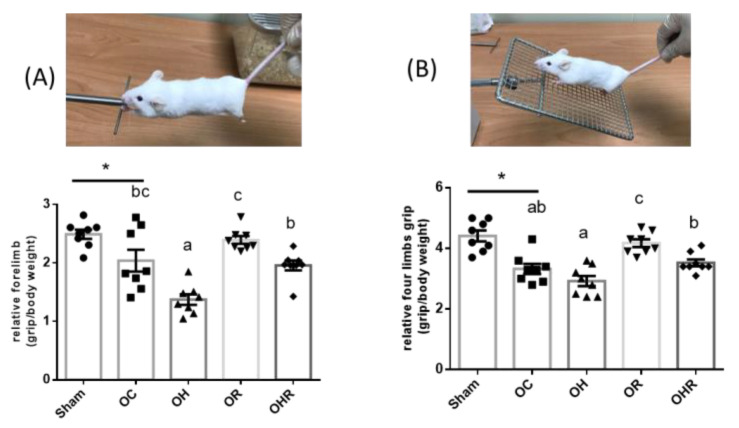
Forelimb (**A**) and four-limb (**B**) grip strength of the study groups. Data are presented as mean ± SEM (*n* = 8). * Significant differences between the OC and sham groups (*p* < 0.05). Different letters indicate significant differences between the OC, OH, OR, and OHR groups (*p* < 0.05).

The relative forelimb grip was significantly lower in the OC group than in the sham group, significantly lower in the OH group than in the OC group, and significantly higher in the OHR group than in the OH group (Figure 3A). The relative four-limb grip was significantly lower in the OC group than in the sham group and significantly higher in the OHR group than in the OH group (Figure 3B). Therefore, rice bran supplementation increased muscle strength in the HFD-fed OVX mice.

### 3.5. The Proinflammatory Cytokine and GLUT4 mRNA Expression in Gastrocnemius Muscle

**Figure 4:**
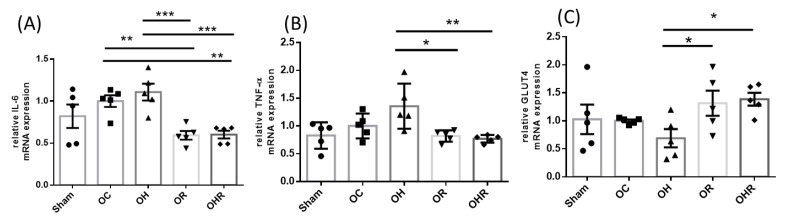
mRNA expression levels of cytokines and GLUT 4 in skeletal muscle of the study groups. mRNA expression levels of (**A**) IL-6, (**B**) TNF-α, and (**C**) GLUT4. Data are presented as mean ± SEM (*n* = 5). * *p* < 0.05; ** *p* < 0.01 and *** *p* < 0.001 was reported with statistical difference between groups.

The mRNA expression level of proinflammatory cytokines IL-6 and TNF-α was significantly lower in the OHR group than in the OH group (Figure 4A,B). The mRNA level of GLUT4 was significantly higher in the OHR group than in the OH group (Figure 4C). Therefore, rice bran supplementation downregulated the expression of proinflammatory cytokines and promoted glucose transport to cell membranes in HFD-fed OVX mice.

### 3.6. Expression Levels of Biomarkers Associated with Synthesis and Breakdown of the Gastrocnemius Muscle of the Mice

**Figure 5:**
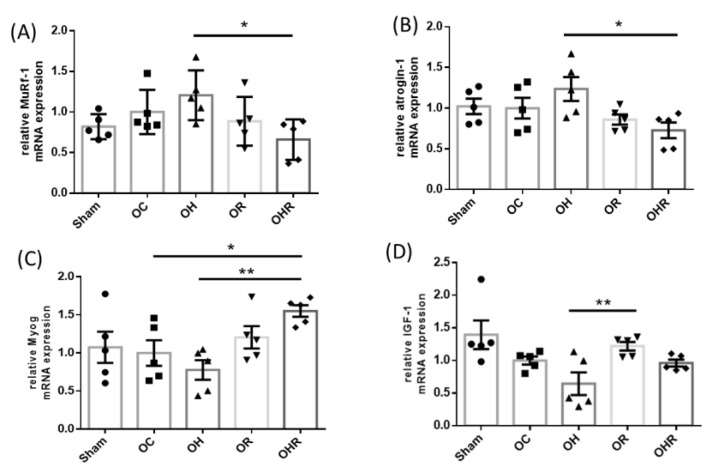
Expression levels of various factors assoiated with skeletal muscle atrophy and myogenesis in the study groups. mRNA expression level of (**A**) MuRF-1, (**B**) atrogin-1, (**C**) Myog, and (**D**) IGF-1. Data are presented as mean ± SEM (*n* = 5). * *p* < 0.05; ** *p* < 0.01 was reported with statistical difference between groups.

The mRNA expression levels of muscle atrophy factors atrogin-1 and MuRF-1 were significantly lower in the OHR group than in the OH group (Figure 5A,B). The mRNA levels of the muscle synthesis factor Myog were significantly higher in the OHR group than in the OH group (Figure 5C). The mRNA expression of IGF-1 was nonsignificantly higher in the OR group than in the OC group and in the OHR group than in the OH group. (Figure 5D). Therefore, rice bran supplementation reduces the incidence of muscle atrophy.

### 3.7. Protein Expression Levels of Muscle Atrophy-Related Biomarkers

**Figure 6:**
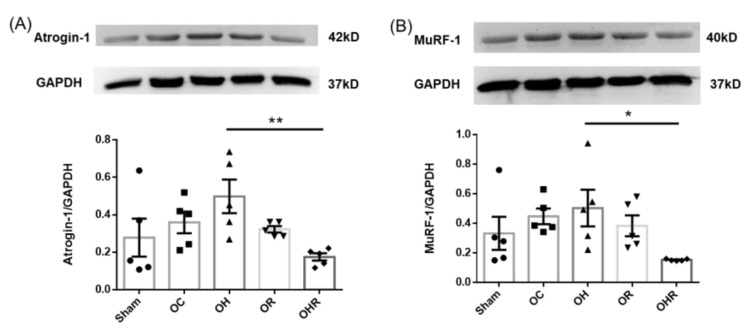
The muscle atrophy (**A**) MuRf-1 and (**B**) atrogin-1 protein expressions of skeletal muscle of mice. Data are presented as mean ± SEM (*n* = 5). * *p* < 0.05; ** *p* < 0.01 was reported with statistical difference between groups.

Atrogin-1 and MuRF-1 are muscle atrophy-related biomarkers. The results showed that the protein expression levels in both atrogin-1 and MuRF-1 were significantly lower in the OHR group than in the OH group (Figure 6). These results are consistent with those of mRNA gene expression.

### 3.8. Gut Microbiota

**Figure 7:**
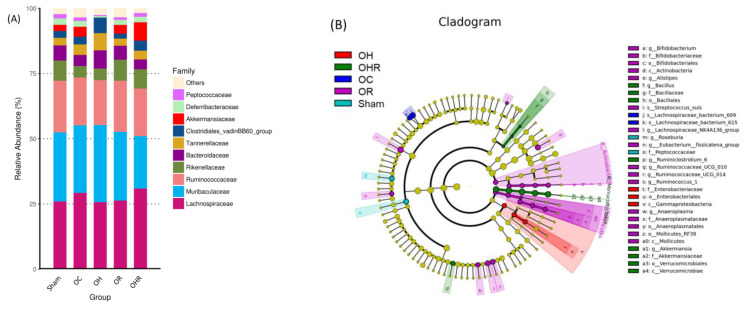

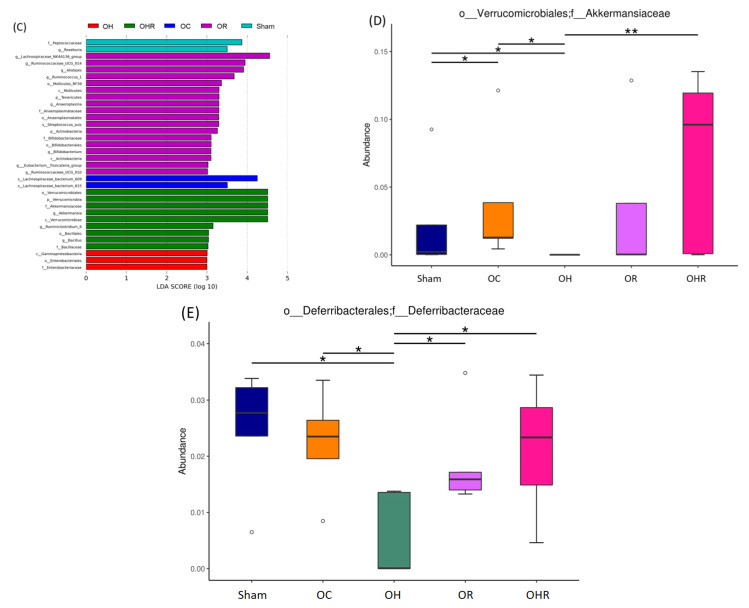
Gut microbiota profiles of the study groups. (**A**) Taxonomic distribution at the family level. (* *p* < 0.05; *n* = 5). (**B**) Linear discriminant analysis(LDA) effect size cladogram. The green color indicates the taxa enriched in OHR group, and the purple color indicates the taxa enriched in OR group (*n* = 5). (**C**) Histogram of the LDA scores. Abundances of (**D**) *Akkermansiaceae* and (**E**) *Deferribacteraceae* at the family level. (* *p* < 0.05, ** *p* < 0.01; *n* =5).

Regarding the effect of rice bran supplementation on the gut microbiota of the experimental mice, at the family level, the abundances of *Akkermansiaceae* and *Deferribacteraceae* were significantly higher in the OHR group than in the OH group (Figure 7A). A linear discriminant analysis effect size cladogram was constructed to depict (from inside to outside) phylum to genus level. The cycle size indicated an abundance of microbiota (Figure 7B). In this study, we set the linear discriminant analysis score at >3, its presence superiority biomarker with the statistical difference between the groups (Figure 7C). Furthermore, the abundance of *Akkermansiaceae* and *Deferribacteraceae* at the family level was significantly higher in the OHR group than in the OH group (Figure 7D,E). These results showed that rice bran intervention for twelve weeks altered the composition of gut microbiota of the mice, particularly ameliorating the dysbiosis in HFD-fed OVX mice.

### 3.9. Short-Chain Fatty Acid Profiles in the Feces of the Study Groups

**Figure 8:**
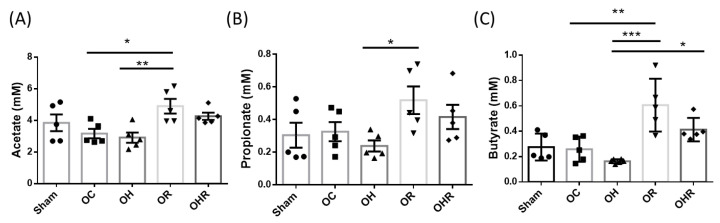
The concentration of SCFAs in feces of study groups; (**A**) Acetate (**B**) Propionate (**C**) Butyrate concentration. Data are presented as mean ± SEM (*n* = 5). * *p* < 0.05; ** *p* < 0.01 and *** *p* < 0.001 was reported with statistical difference between groups.

The concentration of acetate in feces was significantly higher in the OR group than in the OC group (Figure 8A). The concentration of acetate, propionate, and butyrate were significantly higher in the OR group than in OH group (Figure 8). In butyrate, the result showed that the concentration was significantly higher in OHR group than in the OH group (Figure 8C). Therefore, rice bran supplementation increased the butyrate production in the feces of mice.

### 3.10. Expression Levels of Tight Junction Markers of the Colon of the Mice

**Figure 9:**
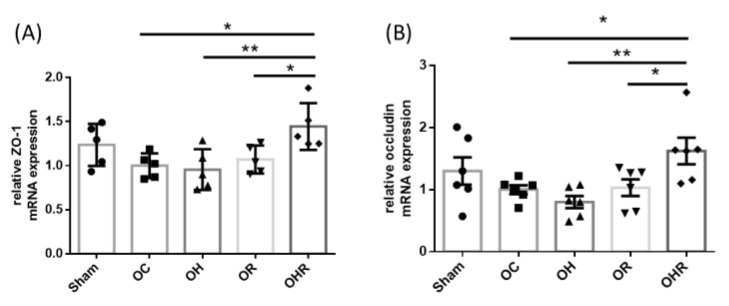
mRNA expression levels of (**A**) occludin, (**B**) ZO-1 of the colon of the study groups. Data are presented as mean ± SEM (*n* = 5). * *p* < 0.05; ** *p* < 0.01 was reported with statistical difference between groups.

The occludin and ZO-1 mRNA expression levels in the OHR group were significantly higher than in the OH group (Figure 9A,B). Therefore, rice bran supplementation improved the gut tight junction genes in HFD-fed OVX mice.

## 4. Discussion

Dietary fatty acid composition is complex and rarely obtained from a single source of dietary lipids. Thus, we used approximately half of the soybean oil and half of the lard content in the high-fat diet to simulate the fat composition of animal- and plant-based diets in daily meals. For rodents, a regular diet contains about 10% fat, so both 45 and 60% fat of the total calorie diet could be served as the HFD [23]. Some studies fed animals a high-fat diet with 40% fat of the total calories [24,25]. A postmenopausal study also fed OVX mice with an HFD containing 40% fat of the total calorie [26]. Thus, based on the fat content of the diet from these studies, we used 40% fat of the total calories as a high-fat diet for the present study.

Estrogen plays an important role in adipocyte differentiation and body fat distribution [27]. A low estrogen level is associated with an increased risk of obesity in postmenopausal women. The postmenopausal loss of estrogen can be mimicked in OVX animal models. We also established an OVX model with a menopause condition in this study. The results revealed a significant reduction in the plasma level of E2, and the weight of uterus in OVX mice. A relevant study reported significantly increased body weight and reduced uterine weight in female C57BL/6 mice after ovariectomy [28]. In the present study, the body weight of the mice significantly increased in the OC group, suggesting that body weight increases after menopause. In another study, gastrocnemius muscle weight was significantly lower in OVX rats than in sham rats [29]. In our study, the relative muscle mass was significantly lower in the OC group than in the sham and OR groups. Moreover, the relative muscle mass was significantly higher in the OHR group than in the OH group. These findings revealed that rice bran supplementation prevents muscle loss under menopausal conditions. Gut microbiota may ferment dietary fiber in the intestine to produce SCFAs. Among the SCFAs, butyric acid helps maintain muscle mass by regulating anti-inflammatory responses, increasing ATP production, and inhibiting muscle breakdown and apoptosis [30]. Our finding indicated that rice bran supplementation contributed to maintaining muscle mass. This may be because rice bran contains high levels of dietary fibers, thus promoting SCFA production and thereby preventing muscle loss.

In a relevant study, C57BL/6 mice fed with an HFD (fat accounting for 50% of the total calories) and rice bran (300 mg/kg/day) exhibited reductions in body weight and relative adipose tissue weight [31]. Another study indicated that male ICR mice treated with 100 mg/kg γ-oryzanol for three days before an HFD (fat accounting for 45% of the total calories) had significantly lower body weight than the mice fed with only an HFD [32]. In the present study, rice bran supplementation reduced weight gain in the HFD-fed OVX mice, likely because of its γ-oryzanol content. 

Postmenopausal women experience lipid profiles abnormalities such as increased blood TC and TG [33]. In a study on HFD-fed rats, the HFD induced increases in the plasma TC, TG, and BUN [34]. Our findings revealed that adding rice bran to the HFD reduced the TG and BUN levels in the OVX mice. Rice bran reduced the plasma glucose in an HFD-fed Sprague–Dawley rats model [35]. Another study reported that HFD (45% of the total calorie) intake combined with 0.05% ferulic acid or 0.16% γ-oryzanol treatment reduced the glucose AUC, glucose level, insulin level, and HOMA-IR value in Sprague–Dawley rats [36]. A rice bran fiber diet reduced postprandial blood glucose levels in type 2 diabetic patients [37]. A study reported that rice bran fractions had a beneficial effect on blood pressure and glucose metabolism in spontaneously hypertensive rats [38]. In the present study, we treated OVX ICR mice with an HFD (40% of the total calorie) combined with 10% (*w*/*w*) rice bran which containing 115 μg/g ferulic acid and 119.1 μg/g γ-oryzanol; after 12 weeks, we observed reductions in blood glucose-related biomarkers, glucose AUC, glucose level, insulin level, and the HOMA-IR value. We speculate that this led to a reduction in the levels of glucose-related biomarkers, which can be attributed to the presence of active components such as ferulic acid and γ-oryzanol, as well as the high dietary fiber content in rice bran.

With increasing age, the leg muscle mass gradually declines; in women, this decline occurs rapidly at around 55 years of age in women [39,40]. A study reported on the grip strength of OVX mice performed in week 24 of the experiment; the muscle strength of OVX mice was significantly lower than the sham control group [41]. Another study reported that the relative grip strength was significantly lower in C57/BL6 OVX mice than in control C57/BL6 mice [42]. Consistent with these findings, our results suggest that both forelimb and four-limb grip strength was significantly lower in the OC group than in the sham group, indicating that reduced muscle strength occurred after ovariectomy surgery. To the best of our knowledge, our study is the first to validate that an HFD results in reduced forelimb grip strength in OVX mice, and that muscle strength increases after rice bran supplementation.

Aging and estrogen deficiency lead to a loss of muscle mass. Aging-related protein decomposition is primarily dependent on the ubiquitination of the ubiquitin–proteasome system. Atrogin-1 and MuRF-1 are essential and specific E3 ubiquitin ligases in skeletal muscle. Their expression is significantly upregulated during muscle atrophy. Given that they are expressed almost exclusively in skeletal muscle, they may serve as biomarkers of muscle atrophy [43]. A study showed that an HDF induced skeleton muscle wasting [44]. The intake of an HFD exacerbates proinflammatory responses and promotes muscle protein degradation, resulting in muscle loss [45]. The expression of the muscle atrophy-related factors atrogin-1 and MuRF-1 is upregulated, resulting in muscle loss. In male BALB/c mice fed with an HFD (60% of the total calorie) for ten weeks; the expression of atrogin-1 and MuRF-1 was upregulated [46], suggesting that an HFD intake leads to muscle loss. Another study reported that male C57BL/6 mice fed with an HFD for 9 weeks exhibited significantly higher expression levels of muscle atrophy-related factors atrogin-1 and MuRF-1 than in control mice. Furthermore, the expression of the proinflammatory cytokine TNF-α was upregulated [47]. Our finding also revealed that HFD intake significantly reduced muscle mass and increased atrogin-1 and MuRf-1 gene expression and proinflammatory cytokine levels. Ferulic acid is found in rice endosperm, wheat bran, barley grain, corn bran, and other plant cell walls. It is a common polyphenol with low toxicity and anti-inflammatory antioxidant properties [48,49]. In the muscle degradation pathway, proinflammatory cytokines (e.g., TNF-α) can activate nuclear factor (NF)-κB, thus promoting the atrogin-1 and activating MuRF1, which results in the loss of skeletal muscle [50]. In our study, the mRNA expression levels of the muscle atrophy-related factors (atrogin-1 and MuRF-1) and proinflammatory cytokines IL-6 and TNF-α were significantly reduced in the OHR groups. In this HFD menopausal animal model, rice bran reduced muscle atrophy by downregulating the expression of muscle atrophy-related factors and proinflammatory cytokines.

Gut microbes play vital roles in the regulating of host metabolism in both human and animal studies [51]. These microbiomes may affect the skeletal muscle metabolism through several pathways. The gut microbiota–muscle axis may be associated with the Toll-like receptor/NF-κB pathway. The abundance of *Akkermansia muciniphila* (*A. muciniphila*) has been reported to correlate inversely with obesity and diabetes in both mice and humans [52,53,54]. A study reported that an HFD (containing 60% calories from fat) induced mice obesity; the relative abundance of *Akkermansia* in feces was lower than in mice fed with a normal control diet [55]. The abundance of *Akkermansia* in the cecum content of mice was lower in HFD-fed OVX mice than in the OC group in this study. These findings indicate that a high-fat diet leads to a decrease in the abundance of *Akkermansia* in the gut, consistent with the previous study. However, there were no significant differences between the OR and OHR groups. Many factors in the OHR group affected animals, including ovariectomy surgery, high-fat diet, and rice bran; thus, the individual differences were higher in OHR group. *A. muciniphila* supplementation reduced body weight, plasma lipopolysaccharide level, and insulin resistance in C57BL/6 mice with diet-induced obesity [56]. The oral administration of *A. muciniphila* prevents HFD induced obesity by altering the adipose tissue metabolism and gut barrier function [53]. Male C57BL/6 mice fed with an HFD supplemented with *A. muciniphila* reduced fat accumulation and promoted muscle cells to increase glucose utilization for reducing insulin resistance [57]. The HFD-induced alterations in gut microbiota profiles increases intestinal permeability by downregulating the expression of two TJS proteins, ZO-1 and occludin [58]. *A. muciniphila* can modulate the expression of ZO-1 and occludin in the intestine and reduce the permeability of the intestinal epithelial barrier [59]. Our finding indicated that the abundance of *Akkermansiaceae* was significantly reduced in the OH group. However, in the OHR group, the abundance of *Akkermansiaceae* increased significantly. It may reduce insulin resistance, perhaps because rice bran contains a high level of dietary fiber. C57BL/6 mice fed with a 60% (*w*/*w*) HFD for 20 weeks exhibited an increased abundance of *Deferribacteraceae* [44]. By contrast, in the present study, the abundance of *Deferribacteraceae* was lower in the OH group than in the OC group. Dietary fiber from rice bran benefits host health by regulating the gut microbiota. Dietary fiber can increase the production of some beneficial metabolites during fermentation, especially SCFAs [60]. A study showed that intake of butyrate or the butyrate-producing bacteria *Akkermansia* spp. has beneficial effects on body weight management [61]. In the present study, the butyrate concentration and the abundance of *Akkermansiaceae* increased in the OHR group, with less weight gain. This may be caused by the dietary fiber of rice bran affecting the gut microbiota and increasing its metabolite butyric acid production.

The intestinal TJs proteins, such as occludin and ZO-1, are crucial for maintaining the integrity of epithelial barrier integrity [13]. Their expression was downregulated in HFD-fed OVX mice. HFD intake has been demonstrated to induce TJ disruption in a type 2 prediabetes model [62]. This may occur through the downregulation of expression of ZO-1, leading to obesity and insulin resistance. Our findings revealed that rice bran supplementation upregulated the expression of occludin and ZO-1 in the OHR group, thereby reversing the HFD-induced increase in intestinal permeability.

The limitation of this study was that the expression of muscle-related biomarkers was similar between the sham and OC groups due to the age of the mice being close to the timing of the premenopausal stage, and a young control may be needed for comparison. In addition, there may be individual differences in the gut microbiota. Since the animals used in the present study were not inbred mice and were not housed in a germ-free environment, these factors may also interfere with the performance of gut microbiomes. We conducted the muscle atrophy of this research using an HFD-fed OVX animal model, so the degree of muscle atrophy may not be as severe as severe as in a dexamethasone-induced animal model [63].

## 5. Conclusions

Our finding indicated that rice bran supplementation reduced weight gain in HFD-fed OVX mice and minimized muscle loss by downregulating the expression of muscle atrophy factors atrogin-1 and MuRF1. Moreover, rice bran increased gut microbiome diversity and abundance and helped maintain better gut barrier function in OVX mice. This research provided beneficial evidence of rice bran supplementation for gut microbiota and muscle atrophy in postmenopausal conditions.

## Figures and Tables

**Table 1:** Body weight, food intake, and energy consumption of the study groups.

Item	Sham	OC	OH	OR	OHR
Initial body weight (g)	39.49 ± 0.31	38.81 ± 0.61	36.73 ± 0.54	38.38 ± 0.61	37.18 ± 0.57
Final body weight (g)	43.19 ± 0.75	53.94 ± 2.53 *^ab^	58.58 ± 2.75 ^b^	45.77 ± 1.56 ^a^	48.41 ± 1.73 ^a^
Weight gain (g)	3.70 ± 0.99	15.13 ± 2.24 *^bc^	21.85 ± 2.35 ^c^	7.39 ± 1.52 ^a^	11.24 ± 1.57 ^ab^
Food intake (g/day)	4.41 ± 0.20	4.02 ± 0.16 *^c^	3.19 ± 0.21 ^a^	3.67 ± 0.16 ^bc^	3.01 ± 0.16 ^a^
Energy (kcal/day)	16.8 ± 0.35	15.11 ± 0.27 *^b^	14.79 ± 0.38 ^ab^	13.96 ± 0.23 ^a^	13.93 ± 0.29 ^a^

Data are presented as mean ± SEM. * Significant differences between the OC and sham groups (*p* < 0.05). Different letters indicate significant differences between the OC, OH, OR, and OHR groups (*p* < 0.05).

**Table 2:** The relative tissue weight of mice.

Tissue	Sham	OC	OH	OR	OHR
	% of Body Weight				
Liver	3.65 ± 0.06	3.65 ± 0.10 ^ab^	3.82 ± 0.20 ^b^	3.58 ± 0.10 ^ab^	3.23 ± 0.12 ^a^
Perinephric adipose tissue	3.81 ± 0.12	6.55 ± 0.54 *	7.40 ± 0.65	5.79 ± 0.30	6.97 ± 0.39
Uterus adipose tissue	2.74 ± 0.16	3.36 ± 0.50 ^a^	6.16 ± 0.69 ^c^	2.79 ± 0.27 ^a^	5.17 ± 0.42 ^bc^
Uterus	0.44 ± 0.06	0.12 ± 0.01 *	0.08 ± 0.01	0.12 ± 0.02	0.10 ± 0.01
Colon	1.56 ± 0.04	1.42 ± 0.10 ^bc^	1.02 ± 0.09 ^a^	1.60 ± 0.09 ^c^	1.20 ± 0.06 ^ab^
Muscle	1.36 ± 0.06	0.71 ± 0.06 *^ab^	0.66 ± 0.04 ^a^	0.89 ± 0.02 ^c^	0.85 ± 0.03 ^bc^

Data are presented as mean ± SEM. * Significant differences between the OC and sham groups (*p* < 0.05). Different letters indicate significant differences between the OC, OH, OR, and OHR groups (*p* < 0.05).

**Table 3:** Blood biochemical profiles.

	Sham	OC	OH	OR	OHR
Estradiol (pg/mL)	7.56 ± 0.45	4.63 ± 0.28 *	4.74 ± 0.42	4.63 ± 0.34	4.63 ± 0.34
Glucose (mg/dL)	152.63 ± 7.16	158.11 ± 8.45 ^bc^	168.88 ± 8.82 ^c^	132.33 ± 4.59 ^a^	137.00 ± 3.79 ^ab^
AST (U/L)	115.71 ± 5.99	113.89 ± 7.74 ^bc^	119.25 ± 8.74 ^c^	88.00 ± 6.13 ^a^	76.63 ± 3.05 ^a^
ALT (U/L)	45.43 ± 10.13	39.44 ± 3.50 ^bc^	43.75 ± 5.00 ^c^	27.11 ± 1.35 ^a^	34.75 ± 1.80 ^abc^
TC (mg/dL)	108.75 ± 5.00	150.67 ± 8.83 *	173.25 ± 9.7	164.00 ± 7.09	181.00 ± 11.10
TG (mg/dL)	88.13 ± 9.09	123.67 ± 4.32 *^c^	117 ± 6.80 ^bc^	119.56 ± 5.14 ^bc^	94.00 ± 6.28 ^a^
BUN (mg/dL)	15.53 ± 0.72	16.81 ± 0.68 ^bc^	17.08 ± 0.87 ^c^	13.48 ± 0.42 ^a^	14.11 ± 0.43 ^a^

Data are presented as mean ± SEM (*n* = 8). * Significant differences between the OC and sham groups (*p* < 0.05). Different letters indicate significant differences between the OC, OH, OR, and OHR groups (*p* < 0.05).

## Data Availability

The data used to support the findings of this study are included within the article.

## Supplementary Materials

The following supporting information can be downloaded at: https://www.mdpi.com/article/10.3390/nu15163514/s1, Table S1: Composition of experimental diet; Table S2: Primers used for the quantitative PCR.
