# Supplementary material for: Rice Bran Supplementation Ameliorates Gut Dysbiosis and Muscle Atrophy in Ovariectomized Mice Fed with a High-Fat Diet
Source: Nutrients. 2023 Aug 9;15(16):3514. doi: 10.3390/nu15163514 (PMC10458250; doi:10.3390/nu15163514)
Supplement: Supplementary file 1 [file nutrients-15-03514-s001.zip › nutrients-2521427-supplementary.pdf]

Table S1 Nutrients composition of experimental diets

Ingredient	Sham	OC	OH	OR	OHR
	g/kg	g/kg	g/kg	g/kg	g/kg
Rice bran[1]	0	0	0	100	100
Maltodextrin	155	155	155	155	155
Sucrose	100	100	100	89.9	89.9
Casein	140	140	140	121.5	121.5
L-Cysetine	2	2	2	2	2
Fresh soybean oil	40	40	104	30.3	94.3
Lard	0	0	96	0	96
Cellulose	50	50	50	15.4	15.4
Mineral-MIX[2]	35	35	35	22.2	22.2
Vitamin-MIX[3]	10	10	10	9	9
Choline Bitartrate	3	3	3	2.75	2.75
Tert-butylhydroquinone	0.008	0.008	0.008	0.008	0.008
% Energy and their source					
Protein	14.9%	14.9%	12.3%	14.9%	12.3%
FAT	9.5%	9.5%	40%	9.5%	40%
Carbohydrate	75.6%	75.6%	47.7%	75.6%	47.7%

1.Rice bran contained 2.3% water; 18.5% protein; 10.1% sucrose; 9.7% fat, 12.8% ash; 34.6% dietary fiber. The experimental diet composition had been adjusted according to the nutrients containing in rice bran.

2. AIN 93 M mineral mixture: Calcium Carbonate 35.7%; Monopotassium phosphate 25.0%; Potassium Citrate monohydrate 2.8%; Sodium Chloride 7.4%; Potassium Sulfate 4.66% Magnesium Oxide 2.4%; Ferric Citrate 0.606%; Zinc Carbonate 0.165%; Manganese Carbonate 0.063%; Copper Carbonate 0.03%; Potassium Iodate 0.001%; Sodium Selenate, Anhydrous 0.001025%; Ammonium Molybdate.4H_2O 0.000795% ;odium Metasilicate.9H_2O 0.145%; Chromium Potassium Sulfate.12H_2O 0.0275%; Lithium Chloride 0.00174%; Boric Acid 0.00815%; Sodium Fluoride 0.00635% ;Nickel Carbonate 0.00318%; Ammonium Vanadate 0.00066% Powdered Sugar 20.9806%.

3. AIN 93 M vitamin mixture: Nicotinic Acid 3.00 (mg/g); D-Calcium Pantothenate 1.60(mg/g);Pyridoxine HCl 0.70(mg/g)Thiamine HCl 0.60(mg/g); Riboflavin 0.60(mg/g) Folic Acid 0.20(mg/g); D-Biotin 0.02(mg/g); Vitamin B12 2.50(mg/g); a-Tocopherol Powder 30.00(mg/g); Vitamin A Palmitate (250,000 U/gm) 1.60(mg/g); Vitamin D3 (400,000 U/gm) 0.25(mg/g); Phylloquinone 0.075(mg/g)Powdered Sucrose 959.655(mg/g).

Table S2 Primers used for the quantitative PCR

	Forward 5′→3′	Reverse 5′→3′
IGF-1	CAATACAGCCAACGGGAAACAG	AACAAAGCTGGATGCCTGTCA
Atrogin	AACCGGGAGGCCAGCTAAAGAACA	TGGGCCTACAGAACAGACAGTGC
MuRF1	CCTTGAGGGCCATTGACTTTG	TCCCCTCAGAACTCAAGAGGAA
Myog	AGTGAATGCAACTCCCACAG	AGTGAATGCAACTCCCACAG
GLUT4	ATTGCAGCGCCTGAGTCTTT	GAGGGGGTTCCCCATCCTTA
IL-6	TCCTACCCCAACTTCCAATGCTC	TTGGATGGTCTTGGTCCTTAGCC
TNF-α	ATGGCCTCCCTCTCATCAGT	TTTGCTACGACGTGGGCTAC
Occludin	ACTGGGTCAGGGAATATCCA	TCAGCAGCAGCCATGTACTC
ZO-1	GGCACATCAGCACGATTTCT	CCACAAAAGAAATCCTTTCACACCT
GAPDH	TGAGCATCTCCCTCACAATTTC	GTGCAGCGAACTTTATTGATGC

IGF-1, Insulin-Like Growth Factor-1; MuRF1,muscle RING-finger protein-1;Myog, muscle-specific gene myogenin; ZO-1, zonula occludens-1.
